# Enhancing Vitamin D3 Efficacy: Insights from Complexation with Cyclodextrin Nanosponges and Its Impact on Gut–Brain Axes in Physiology and IBS Syndrome

**DOI:** 10.3390/ijms25042189

**Published:** 2024-02-11

**Authors:** Francesca Uberti, Francesco Trotta, Roberta Cavalli, Rebecca Galla, Fabrizio Caldera, Sara Ferrari, Simone Mulè, Arianna Brovero, Claudio Molinari, Pasquale Pagliaro, Claudia Penna

**Affiliations:** 1 Laboratory of Physiology, Department of Translational Medicine, University of Piemonte Orientale, Via Solaroli 17, 28100 Novara, Italy; francesca.uberti@med.uniupo.it (F.U.); rebecca.galla@uniupo.it (R.G.); sara.ferrari@uniupo.it (S.F.); simone.mule@uniupo.it (S.M.); 2 Dipartimento di Chimica and NIS, Università di Torino, Via P. Giuria 7, 10125 Torino, Italy; fabrizio.caldera@unito.it; 3 Dipartimento di Scienza e Tecnologia del Farmaco, Università di Torino, Via P. Giuria 9, 10125 Torino, Italy; roberta.cavalli@unito.it; 4 Laboratory of Cardiovascular Physiology, Dipartimento di Scienze Cliniche e Biologiche, Università Degli Studi di Torino, Regione Gonzole 10, 10043 Orbassano, Italy; arianna.brovero@unito.it (A.B.); pasquale.pagliaro@unito.it (P.P.); claudia.penna@unito.it (C.P.); 5 Dipartimento per lo Sviluppo Sostenibile e la Transizione Ecologica, University of Piemonte Orientale, 13100 Vercelli, Italy; claudio.molinari@med.uniupo.it; 6 National Institute for Cardiovascular Research (INRC), 40126 Bologna, Italy

**Keywords:** vitamin D, nanosponge, intestinal cells, absorption mechanism, inflammatory bowel syndrome, gut–brain axis

## Abstract

Vitamin D3 (VitD3) plays a crucial role in various cellular functions through its receptor interaction. The biological activity of Vitamin D3 can vary based on its solubility and stability. Thus, the challenge lies in maximizing its biological effects through its complexation within cyclodextrin (βNS-CDI 1:4) nanosponges (NS) (defined as VitD3NS). Therefore, its activity has been evaluated on two different gut–brain axes (healthy gut/degenerative brain and inflammatory bowel syndrome gut/degenerative brain axis). At the gut level, VitD3-NS mitigated liposaccharide-induced damage (100 ng/mL; for 48 h), restoring viability, integrity, and activity of tight junctions and reducing ROS production, lipid peroxidation, and cytokines levels. Following intestinal transit, VitD3-NS improved the neurodegenerative condition in the healthy axis and the IBS model, suggesting the ability of VitD3-NS to preserve efficacy and beneficial effects even in IBS conditions. In conclusion, this study demonstrates the ability of this novel form of VitD3, named VitD3-NS, to act on the gut–brain axis in healthy and damaged conditions, emphasizing enhanced biological activity through VitD3 complexation, as such complexation increases the beneficial effect of vitamin D3 in both the gut and brain by about 50%.

## 1. Introduction

Vitamin D3 (VitD3) is a pivotal player in cellular functions, extending its influence beyond bone health to cardiovascular well-being and intestinal processes through its interaction with the vitamin D receptor (VDR) [1,2,3,4,5]. Despite its physiological significance, solubility and stability challenges hinder optimal administration, raising the question of maximizing VitD3’s potential for overall health [1,2,3,4,5]. To address these challenges, researchers explore innovative strategies to enhance VitD3’s solubility and stability. One approach involves complexing VitD3 with Cyclodextrins (CDs), truncated cone-shaped oligosaccharides derived from starch [6]. This novel strategy aims to make VitD3 more usable, unlocking its therapeutic potential. Despite ongoing efforts, a comprehensive understanding of the efficacy and cellular impact of CD–VitD3 complexes remains a topic of exploration [5]. Our group contributes to this endeavor by delving into the intricate dynamics of CD–VitD3 complexes, emphasizing their impact at the intestinal level. The hypothesis guiding our exploration suggests that CD complexation could offer a viable solution to the persistent solubility and stability challenges associated with VitD3 [5]. Concurrently, recent studies shed light on bidirectional gut–brain interactions regulating key functions, including food intake and immune regulation [7]. Disruptions in the Gut–Brain Axis (GBA) can lead to conditions like irritable/inflammatory bowel syndrome (IBS), implicating the intestinal microbiome [8]. Dietary triggers, such as fermentable oligosaccharides, contribute to IBS symptoms [9]. IBD introduces additional complexity involving lipopolysaccharide (LPS) [10]. Indeed, LPS, at the intestinal level, activates NF-kB, leading to inflammatory cytokine production and increased permeability [9]. This underscores the gut’s critical role beyond gastrointestinal concerns, impacting systemic inflammatory responses. Interestingly, evidence suggests an association between IBS and micronutrient deficiencies, including vitamin D [11]. Low vitamin D levels correlate with various intestinal pathologies [12]. Vitamin D3 influences mucosal immunity, barrier function, and microbial composition [12]. Research by Dussik’s group underscores VitD3’s role in modulating serotonin synthesis and gene expression associated with IBS [13]. This convergence highlights the interconnectedness of micronutrients, like VitD3, with gut health and broader systemic functions.

Our study seeks to bridge the gap between these two realms—the complexities of VitD3 utilization and the intricate dynamics of gut–brain interactions. By elucidating the potential benefits of CD–Vit D3 complexes at the intestinal level, we aim not only to contribute to the understanding of cellular interactions but also to address significant gaps in current knowledge concerning the practical applications of Vitamin D3 in promoting intestinal health. Our research holds the promise of shedding light on the therapeutic potential of VitD3 cyclodextrin nanosponges (VitD3-NS) complexes, offering a novel perspective on enhancing the bioavailability of Vitamin D3 and, consequently, its efficacy in cellular functions, with a particular emphasis on the intestinal context. This approach may uncover new avenues for therapeutic interventions, ultimately improving the well-being of individuals facing challenges in Vitamin D3 utilization and gut–brain interactions.

## 2. Results

### 2.1. Time-Course Study on the Effect of Vitamin D Forms on CaCo-2 Cells Subjected to IBS Conditions

**Figure 1:**
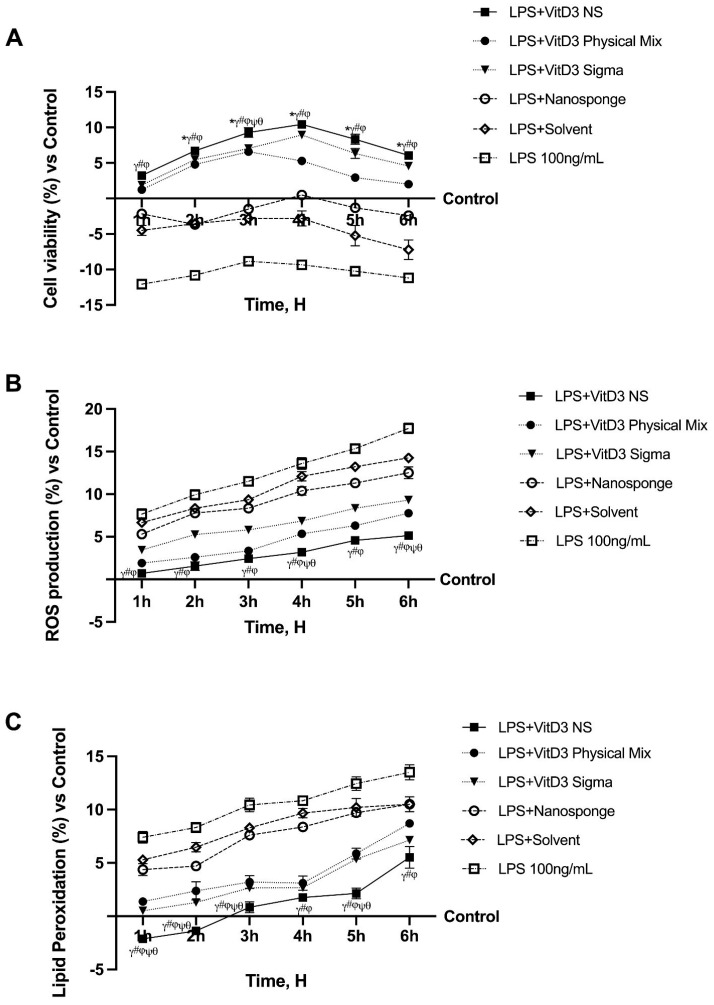
Analysis of different forms of VitD3 in the time-dependent study. In (**A**), cell viability; in (**B**), ROS production; and in (**C**), Lipid Peroxidation analysis. LPS = 100 ng/mL lipopolysaccharide; VitD3 NS = 100 nM Vitamin D3-BCDI 1:4 nanosponge loaded; VitD3 physical Mix = 100 nM Physical Mix Vitamin D3-BCDI 1:4; VitD3 Sigma = 100 nM Vitamin D3 purchased from Merck Life Science; Nanosponge = BCDI (1:4 nanosponge); Solvent = 0.007% Ethanol. Data are expressed as mean ± SD (%) of 5 independent experiments normalized to control. * *p* < 0.05 vs. control; ^γ^ *p* < 0.05 vs. LPS 100 ng/mL; ^#^ *p* < 0.05 vs. solvent (ethanol); ^φ^ *p* < 0.05 vs. nanosponge; ^ψ^ *p* < 0.05 vs. VitD3 Sigma; ^θ^ *p* < 0.05 vs. VitD3 physical Mix 100 nM.

Time-dependent experiments were conducted to evaluate whether the biological efficacy of the VitD3 NS complex remained unaltered in a cell monolayer model under inflammatory conditions. These experiments continue a previous study in which the same cells were tested under physiological conditions [5]. To determine whether this complex could be absorbed intestinally under IBS conditions, CaCo-2 cells previously treated with LPS 100 ng/mL for 48 h before VitD3 100 nm treatment in several forms (such as VitD3 NS, VitD3 Physical Mix, and VitD3 (named VitD3 named Sigma) were used to perform a time-dependent study to exclude any cytotoxic effect. As shown in Figure 1A, cell viability decreases after treatment with LPS 100 ng/mL compared to control (*p* < 0.05), but treatment with VitD3 restores cell viability compared to LPS 100 ng/mL alone. In particular, at 4 h of treatment, VitD3 NS exerts a greater effect of about 1.89-fold than LPS 100 ng/mL and about 49% and 17% compared to VitD3 Physical Mix 100 nM and VitD3 Sigma 100 nM. The vehicle and nanosponges could not restore cell viability to the control level (*p* < 0.05). In addition, as illustrated in Figure 1B, all forms of VitD3 restore oxidative stress to control levels (*p* < 0.05); in particular, VitD3 NS reduces oxidative stress in IBS condition compared to LPS 100 ng/mL, VitD3 Physical Mix 100 nM, and VitD3 Sigma 100 nM (*p* < 0.05). In this case, ROS generation after LPS-induced damage is not restored to control by nanosponges alone and vehicle. To establish the reparative action of VitD3 forms, additional studies were undertaken to assess cellular function and mitochondrial issues. After induction of damage with LPS 100 ng/mL for 48 h, the decrease in MDA was studied over a treatment period from 1 to 6 h with 100 nM VitD3 NS, VitD3 Physical Mix 100 nM, and VitD3 Sigma 100 nM. As shown in Figure 1C, all forms of VitD3 succeed in reducing MDA production compared with LPS-induced damage 100 ng/mL throughout the treatment period; specifically, the greatest ameliorative effect is exerted by VitD3 NS compared to the other two forms of VitD3 Physical Mix 100 nM and VitD3 Sigma 100 nM.

### 2.2. Permeability Analysis of the Effects of Different Vitamin D3 Forms in an In Vitro Intestinal Barrier under IBS Conditions

**Figure 2:**
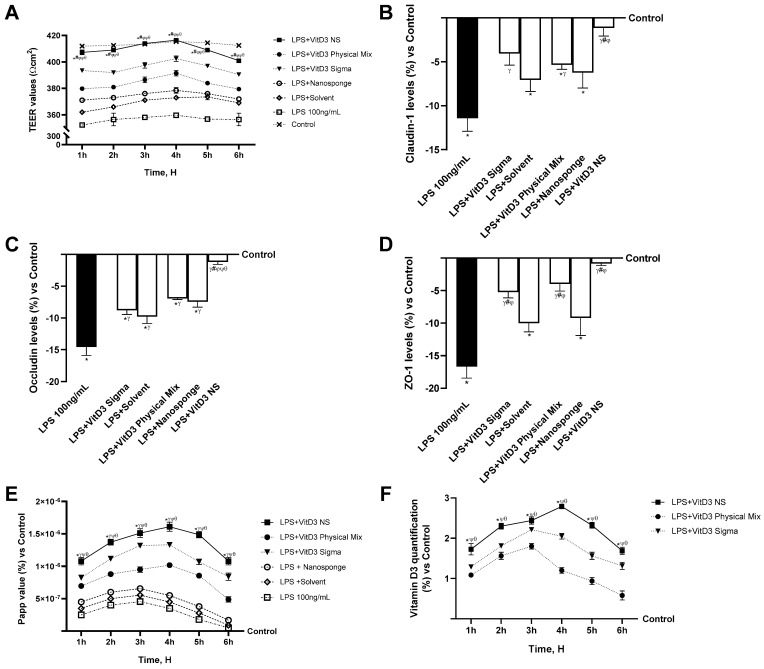
Permeability study on CaCo-2 cells under IBS conditions. In (**A**), TEER (transepithelial electrical resistance) Value using EVOM3; from (**B**–**D**), the analysis of TJ measured by Enzyme-Linked Immunosorbent Assay (ELISA) test (Occludin, Claudin1, and ZO-1 (Zona occludens 1), respectively); in (**E**), the Papp (Apparent Permeability Coefficient) values in which data < 0.2 × 10^−6^ cm/s mean very poor absorption with a bioavailability < 1%, data between 0.2 × 10^−6^ and 2 × 10^−6^ cm/s with bioavailability between 1 and 90%, and data >2 × 10^−6^ cm/s mean very good absorption with a bioavailability over 90%. In (**F**), VitD3 quantification was measured using an ELISA kit. Data are expressed as mean ± SD (%) of 5 independent experiments normalized to control. * *p* < 0.05 vs. control; ^γ^ *p* < 0.05 vs. LPS 100 ng/mL; ^#^ *p* < 0.05 vs. solvent (ethanol); ^φ^ *p* < 0.05 vs. nanosponge; ^ψ^ *p* < 0.05 vs. VitD3 Sigma; ^θ^ *p* < 0.05 vs. VitD3 Physical Mix 100 nM.

Further investigations were conducted by assessing intestinal permeability in a 3D in vitro model that mimics the intestine subjected to IBS conditions to accumulate data on the intestinal absorption of VitD3 NS. Hence, transepithelial electrical resistance (TEER) values, apparent permeability coefficient (Papp) values, and VitD3 concentration were evaluated from 1 to 6 h for VitD3 NS, VitD3 Physical Mix, and VitD3 Sigma at the same concentration to assess bioavailability. As can be seen from the TEER graph (Figure 2A), the 100 ng/mL LPS-induced damage impaired the integrity of the cell monolayer compared to the control (*p* < 0.05), resulting in its perforation. However, despite the obvious damage induced by LPS 100 ng/mL, VitD3 NS restored the damage by reassembling the integrity of the cell monolayer to a greater degree than the other two forms (*p* < 0.05). Moreover, TJs analysis confirmed these results (Figure 2B–D); in particular, VitD3 NS treatment improved TJs levels during the treatment more than LPS 100 ng/mL, VitD3 Physical Mix 100 nM, and VitD3 Sigma 100 nM in terms of claudin-4 (90%, 79%, and 72%, respectively, *p* < 0.05), occludin (92%, 83%, and 86%, respectively, *p* < 0.05), and ZO-1 (95%, 80%, and 85%, respectively, *p* < 0.05) and when compared to the control value (line 0, *p* < 0.05). These encouraging findings certified the normal function of the intestinal epithelium; further experiments were performed by measuring the permeability rate, analyzing the flux of nonelectrolyte tracers (expressed as the permeability coefficient, Figure 2E), and the amount of VitD3 crossing the intestinal barrier tract to reach the target site (Figure 2F).

**Figure 3:**
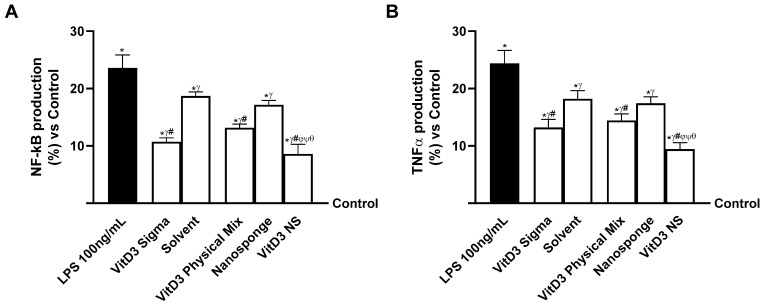
Inflammatory status analysis on CaCo-2 cells. In (**A**), NF-kB analysis measured by Enzyme-Linked Immunosorbent Assay (ELISA) test; and in (**B**), TNFα analysis measured by ELISA kit. Data are expressed as mean ± SD (%) of 5 independent experiments normalized to control. * *p* < 0.05 vs. control; ^γ^ *p* < 0.05 vs. LPS 100 ng/mL; ^#^ *p* < 0.05 vs. solvent (ethanol); ^φ^ *p* < 0.05 vs. nanosponge; ^ψ^ *p* < 0.05 vs. VitD3 Sigma; ^θ^ *p* < 0.05 vs. VitD3 Physical Mix 100 nM.

To confirm the ability of VitD3 forms to ameliorate the gut under IBS conditions, an analysis of NF-kB and TNFα was carried out. As shown in Figure 3, NF-kB and TNFα levels were found to be reduced after treatment with all VitD3 forms compared to LPS 100 ng/mL damage-induced effects (*p* < 0.05). Specifically, VitD3 NS was able to reduce the NF-kB production levels (Figure 3A) better than VitD3 Physical Mix 100 nM and VitD3 Sigma 100 nM (35% and 20%, respectively, *p* < 0.05), reducing the production of damage by approximately 64%. This finding was confirmed by analysis of TNFα production; as shown in Figure 3B, all forms of VitD3 were able to modulate the inflammation process by reducing the response following LPS-induced 100 ng/mL damage (*p* < 0.05). Specifically, VitD3 NS, also in this case, was able to lower TNFα levels better than the other forms of VitD3 (35% vs. VitD3 Physical Mix 100 nM and 29% vs. VitD3 Sigma 100 nM, *p* < 0.05) and was able to reduce inflammation compared to LPS 100 ng/mL by about 61%.

### 2.3. The Effects of VitD3 NS on the Gut–Brain Axis Degeneration Model

**Figure 4:**
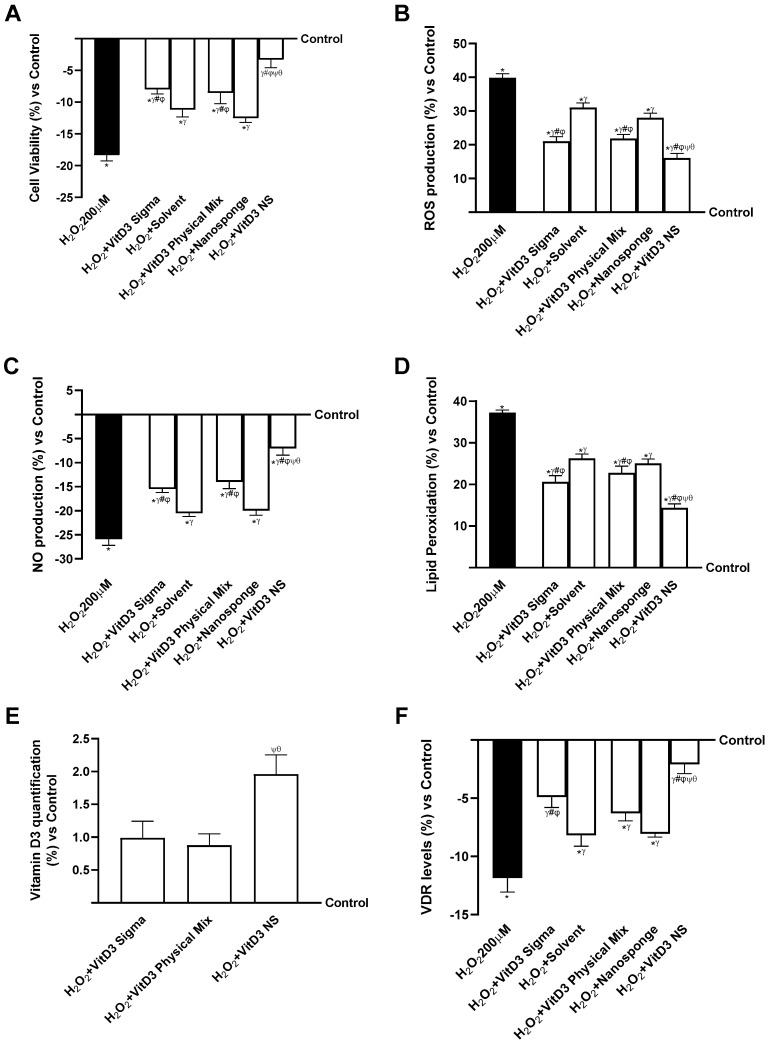
The effects of VitD3 NS on the healthy gut/degenerative brain axis. In (**A**), cell viability is measured by the MTT test; in (**B**), ROS production is measured by Cytochrome C reduction; in (**C**), NO production is measured by a specific Assay Kit; in (**D**), lipid peroxidation analyzed by a specific assay kit; in (**E**), Vitamin D3 quantification measured by ELISA Kit; and in (**F**), VDR level evaluated by ELISA Kit. Data are expressed as mean ± SD (%) of 5 independent experiments normalized to control. * *p* < 0.05 vs. control; ^γ^ *p* < 0.05 vs. H_2_O_2_ 200 µM; ^#^ *p* < 0.05 vs. solvent (ethanol); ^φ^ *p* < 0.05 vs. nanosponge; ^ψ^ *p* < 0.05 vs. VitD3 Sigma; ^θ^ *p* < 0.05 vs. VitD3 Physical Mix 100 nM.

Since the hypothesized target site of VitD3 NS is reported to be the brain, additional experiments were conducted on neuronal cells by constructing two different models of the gut–brain axis: the healthy gut/degenerative brain axis and the IBS gut/degenerative brain axis. As shown in Figure 4 (healthy gut/degenerative brain axis), all VitD3 forms utilized were able to influence the final target, enhancing cell viability and oxidative stress state (*p* < 0.05) after pretreating for 30 min with H_2_O_2_ 200 µM to mimic neurodegenerative conditions. Notably, on the healthy gut–brain axis, VitD3 NS was able to improve cell viability (Figure 4A, healthy gut/degenerative brain axis, *p* < 0.05) compared to the other two VitD3 forms (61% vs. VitD3 Physical Mix 100 nM and 59% vs. VitD3 Sigma 100 nM, *p* < 0.05) and damage induced by H_2_O_2_ 200 µM. This effect was confirmed by the analysis of ROS production (Figure 4B, healthy gut/degenerative brain axis); indeed, VitD3 NS showed the ability to reduce ROS production better than the other forms VitD3 Physical Mix 100 nM and VitD3 Sigma, by about 27% and 24%, respectively, and by about 60% of the ROS production induced by H_2_O_2_ 200 µM. These positive effects were additionally confirmed by analysis of NO production (Figure 4C, healthy gut/degenerative brain axis) and MDA production (Figure 4D, healthy gut/degenerative brain axis). It is possible to observe that all VitD3 forms were able to increase NO levels compared to H_2_O_2_ 200 µM injury by increasing NO production by about 46% VitD3 Physical Mix 100 nM, 40% VitD3 Sigma 100 nM, and 73% VitD3 NS 100 nM. Particularly, the greatest effect was exhibited by VitD3 NS compared with VitD3 Physical Mix 100 nM and VitD3 Sigma 100 nM by about 50% and 55%, respectively (*p* < 0.05). The same beneficial effect of VitD3 NS was observed after analyzing the production of MDA and the degree of lipid peroxidation. As shown in Figure 4D (healthy gut/degenerative brain axis), degenerative damage induced by H_2_O_2_ 200 µM increases lipid peroxidation by about 37% compared to control (*p* < 0.05). Conversely, all forms of VitD3 reduced lipid peroxidation compared to damage (*p* < 0.05). In detail, VitD3 NS reduced lipid peroxidation better than the other forms (37% vs. VitD3 Physical Mix 100 nM and 30% vs. VitD3 Sigma 100 nM, *p* < 0.05). In conclusion, to determine if VitD3 NS may be able to reach the target site and confirm its biological function explication, VitD3 (Figure 4E, healthy gut/degenerative brain axis) and VDR quantifications (Figure 4F, healthy gut/degenerative brain axis) were assessed at the brain level under neurodegenerative conditions. Even in this case, VitD3 NS was able to reach the target site in higher amounts than the other two forms (55% vs. VitD3 Physical Mix 100 nM and 49% vs. VitD3 Sigma 100 nM, *p* < 0.05), increasing VDR level by about 67% and 60% compared with VitD3 Physical Mix 100 nM and VitD3 Sigma 100 nM despite the presence of H_2_O_2_ 200 µM-induced degenerative damage (*p* < 0.05).

**Figure 5:**
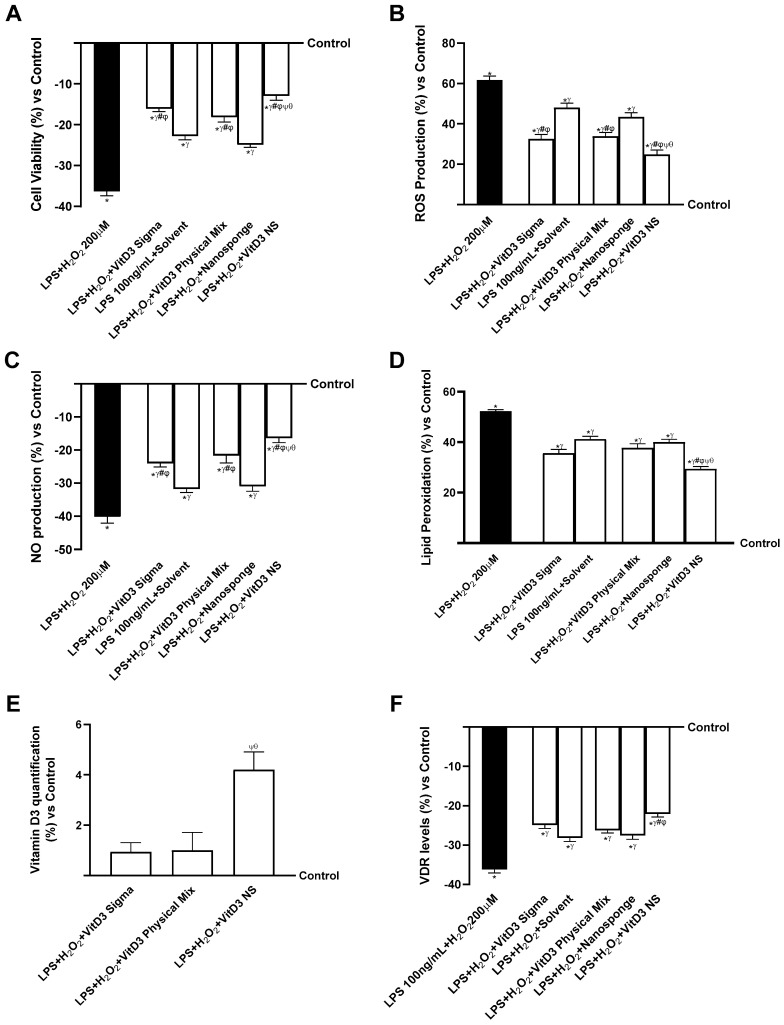
The effects of VitD3 NS on IBS gut/degenerative brain axis. In (**A**), cell viability is measured by the MTT test; in (**B**), ROS production is measured by Cytochrome C reduction; in (**C**), NO production is measured by a specific Assay Kit; in (**D**), lipid peroxidation analyzed by a specific assay kit; in (**E**), Vitamin D3 quantification measured by ELISA Kit; and in (**F**), VDR quantification evaluated by ELISA Kit. Data are expressed as mean ± SD (%) of 5 independent experiments normalized to control. * *p* < 0.05 vs. control; ^γ^ *p* < 0.05 vs. LPS 100 ng/mL + H_2_O_2_ 200 µM; ^#^ *p* < 0.05 vs. solvent (ethanol); ^φ^ *p* < 0.05 vs. nanosponge; ^ψ^ *p* < 0.05 vs. VitD3 Sigma; ^θ^ *p* < 0.05 vs. VitD3 Physical Mix 100 nM.

Further experiments were conducted on neuronal cells under degenerative damage conditions using an IBS gut/brain axis. As shown in Figure 5 (IBS gut/degenerative brain axis), all VitD3 forms could influence the final target despite the condition of IBS and brain degeneration (*p* < 0.05) but without restoring the values to the control level. Notably, on the IBS gut/degenerative brain axis, VitD3 NS was able to improve cell viability (Figure 5A, IBS gut/degenerative brain axis, *p* < 0.05) compared to the other two VitD3 forms (29% vs. VitD3 Physical Mix 100 nM and 20% vs. VitD3 Sigma 100 nM, *p* < 0.05) and damage induced by LPS 100 ng/mL + H_2_O_2_ 200 µM. This effect was confirmed by the analysis of ROS production (Figure 5B, IBS gut/degenerative brain axis); indeed, VitD3 NS showed the ability to reduce ROS production better than the other forms VitD3 Physical Mix 100 nM and VitD3 Sigma by about 27% and 24%, respectively, and by about 60% of the ROS production induced by LPS 100 ng/mL + H_2_O_2_ 200 µM. These positive effects were additionally confirmed by analysis of NO production (Figure 5C, IBS gut/degenerative brain axis) and MDA production (Figure 5D, IBS gut/degenerative brain axis). It is possible to observe that all VitD3 forms were able to increase NO levels compared to LPS 100 ng/mL + H_2_O_2_ 200 µM injury by increasing NO production by about 46% VitD3 Physical Mix 100 nM, 40% VitD3 Sigma 100 nM, and 60% VitD3 NS 100 nM. In particular, the greatest effect was exhibited by VitD3 NS compared with VitD3 Physical Mix 100 nM and VitD3 Sigma 100 nM at about 24% and 32%, respectively (*p* < 0.05). A similar beneficial effect of VitD3 NS was observed after analyzing the production of MDA and the degree of lipid peroxidation. As shown in Figure 5D (IBS gut/degenerative brain axis), degenerative damage induced by LPS 100 ng/mL + H_2_O_2_ 200 µM increases lipid peroxidation by about 52% compared to control (*p* < 0.05). Conversely, all forms of VitD3 reduced lipid peroxidation compared to damage (*p* < 0.05). In detail, VitD3 NS reduced lipid peroxidation better than the other forms (22% vs. VitD3 Physical Mix 100 nM and 17% vs. VitD3 Sigma 100 nM, *p* < 0.05). In conclusion, to determine if VitD3 NS may be able to reach the target site without being altered by intestinal conditions, VitD3 quantification (Figure 5E, IBS gut/degenerative brain axis) and VDR quantifications (Figure 5F, IBS gut/degenerative brain axis) were assessed at the brain level under neurodegeneration conditions. Even in this case, VitD3 NS was able to reach the target site in greater amounts than the other two forms (76% vs. VitD3 Physical Mix 100 nM and 78% vs. VitD3 Sigma 100 nM, *p* < 0.05), increasing VDR level by about 16% and 11% compared with VitD3 Physical Mix 100 nM and VitD3 Sigma 100 nM despite the presence of LPS 100 ng/mL + H_2_O_2_ 200 µM-induced degenerative damage (*p* < 0.05).

### 2.4. Analysis of the Mechanisms Underlying Cognitive Decline on SHSY-5Y Cells in Damaged Conditions

**Figure 6:**
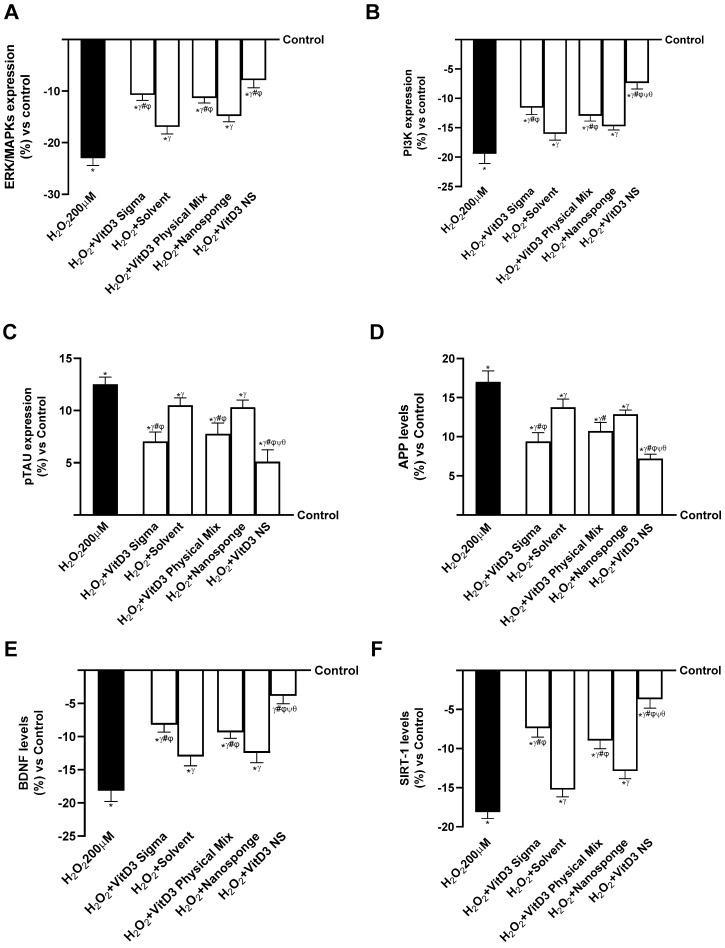
Mechanisms analysis behind cognitive decline on SHSY-5Y cells in damaged condition. In (**A**), ERK/MPAKS expression measured by ELISA Kit; in (**B**), PI3K expression measured by ELISA Kit; in (**C**), pTAU expression measured by ELISA Kit; in (**D**), APP level measured by ELISA Kit; in (**E**), BDNF level measured by ELISA Kit; and in (**F**), SIRT-1 level evaluated by ELISA Kit. Data are expressed as mean ± SD (%) of 5 independent experiments normalized to control. * *p* < 0.05 vs. control; ^γ^ *p* < 0.05 vs. H_2_O_2_ 200 µM; ^#^ *p* < 0.05 vs. solvent (ethanol); ^φ^ *p* < 0.05 vs. nanosponge; ^ψ^ *p* < 0.05 vs. VitD3 Sigma; ^θ^ *p* < 0.05 vs. VitD3 Physical Mix 100 nM.

The ability of different VitD3 forms to prevent cell damage under oxidative conditions was investigated by examining the major intracellular processes that were activated under degenerative conditions following treatment with different forms of VitD3. ERK/MAPK and the phosphatidylinositol-3-kinase (PI3K) pathway are known to play a crucial role in regulating neuronal and brain survival, so further experiments on its expression were conducted in the study. As explained in Figure 6A (healthy gut/degenerative brain axis), treatment of the gut–brain axis with all VitD3 confirmed its potential to increase survivability by activating ERK/MAPK mediators. In addition, VitD3 NS amplified kinase activation compared to H_2_O_2_ 200 µM, VitD3 Physical Mix 100 nM, and VitD3 Sigma 100 nM (63%, 31%, and 27%, respectively, *p* < 0.05). The data shown in Figure 6B (healthy gut–brain axis) also confirmed the ability of VitD3 NS to promote cell survival by increasing PI3K expression better than H_2_O_2_ 200 µM, VitD3 Physical Mix 100 nM, and VitD3 Sigma 100 nM (62%, 43%, and 36%, respectively, *p* < 0.05).

Since the cognitive decline is based on an impairment of neuronal expression, pTAU and β-amyloid (APP) activities were investigated as major markers of cognitive decline, given that their alteration could induce hyperphosphorylation of pTAU and aggregation of APP. As shown in Figure 6C (healthy gut/degenerative brain axis), pTAU expression increased in the presence of H_2_O_2_ 200 µM compared with control (about 12.5%, *p* < 0.05), indicating an involvement of oxidative damage in the alteration of pTAU expression. In contrast, treatment with the different forms of VitD3 significantly reduced pTAU expression compared with H_2_O_2_ 200 µM-induced damage (*p* < 0.05). In particular, the greatest reduction was observed after treatment with VitD3 NS 100 nM (about 59% vs. H_2_O_2_ 200 µM, 34% vs. VitD3 Physical Mix 100 nM, 28% vs. VitD3 Sigma 100 nM, *p* < 0.05), indicating a beneficial effect in counteracting cognitive dysfunction. The positive effect of VitD3 NS was also supported by APP level (Figure 6D, healthy gut/degenerative brain axis); indeed, following treatment with VitD3 NS 100 nM reduced APP level by about 58% vs. H_2_O_2_ 200 µM (*p* < 0.05), about 33% vs. VitD3 Physical Mix 100 nM (*p* < 0.05), and about 23% vs. VitD3 Sigma 100 nM (*p* < 0.05). These findings support previous findings, indicating that VitD3 NS may enhance cell survival through the gut–brain axis mechanism.

In conclusion, the effects of VitD3 NS on SIRT-1 and BDNF levels were studied. As shown in Figure 6E (healthy gut/degenerative brain axis), BDNF level was significantly increased by VitD3 NS 100 nM (*p* < 0.05) compared to the other VitD3 forms (59% vs. VitD3 Physical Mix 100 nM and 53% vs. VitD3 Sigma 100 nM, *p* < 0.05). In addition, as illustrated in Figure 6F (healthy gut/degenerative brain axis), the phosphorylation of SIRT1 induced by VitD3 NS (*p* < 0.05) was increased compared to the other forms of VitD3 (59% vs. VitD3 Physical Mix 100 nM and 50% vs. VitD3 Sigma 100 nM, *p* < 0.05), supporting the efficacy of VitD3 NS in increasing the presence of this molecule.

**Figure 7:**
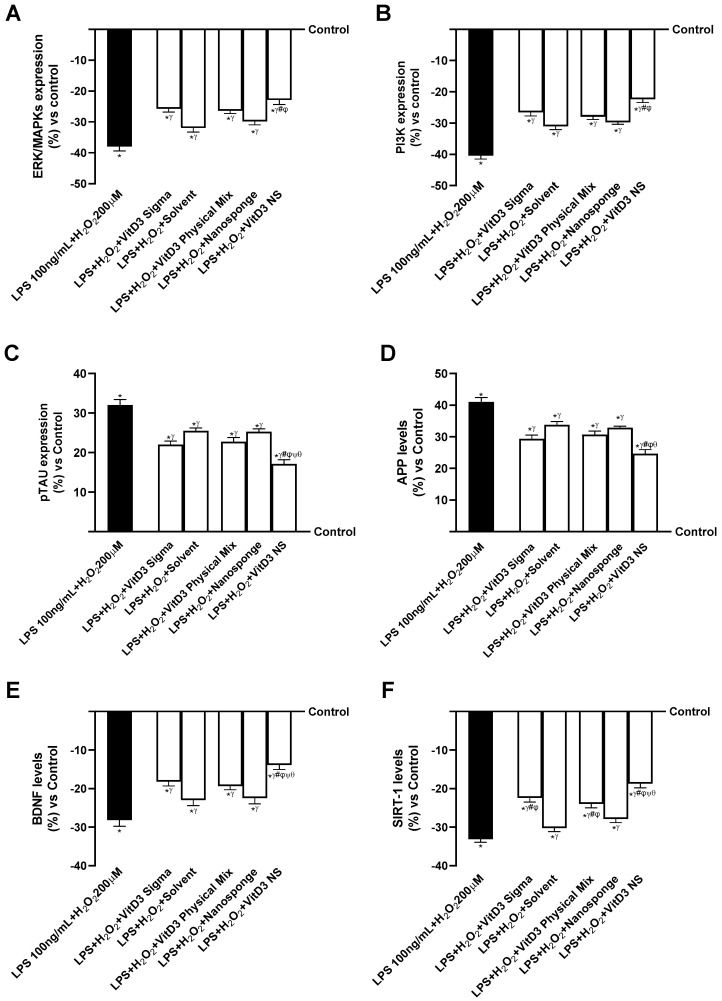
Mechanisms analysis behind cognitive decline on SHSY-5Y cells in damaged condition. In (**A**), ERK/MPAKS expression measured by ELISA Kit; in (**B**), PI3K expression measured by ELISA Kit; in (**C**), pTAU expression measured by ELISA Kit; in (**D**), APP level measured by ELISA Kit; in (**E**), BDNF level measured by ELISA Kit; and in (**F**), SIRT-1 level evaluated by ELISA Kit. Data are expressed as mean ± SD (%) of 5 independent experiments normalized to control. * *p* < 0.05 vs. control; ^γ^ *p* < 0.05 vs. LPS 100 ng/mL + H_2_O_2_ 200 µM; ^#^ *p* < 0.05 vs. solvent (ethanol); ^φ^ *p* < 0.05 vs. nanosponge; ^ψ^ *p* < 0.05 vs. VitD3 Sigma; ^θ^ *p* < 0.05 vs. VitD3 Physical Mix 100 nM.

The IBS gut/degenerative brain axis model investigations were repeated to validate the action on important intracellular neuronal markers. As previously cited, ERK/MAPKs and PI3K are important signaling pathways for neuronal cell survival. Therefore, as shown in Figure 7A,B (IBS gut/degenerative brain axis), the expressions of ERKs/MAPKs and PI3Ks were reduced in the cells treated with injury (LPS 100 ng/mL + H_2_O_2_ 200 µM). In contrast, these activities increased in the cells that received treatment with different forms of VitD3. In addition, VitD3 NS amplified ERK/MAPKs expression compared to LPS 100 ng/mL + H_2_O_2_ 200 µM, VitD3 Physical Mix 100 nM, and VitD3 Sigma 100 nM (40%, 13%, and 11%, respectively, *p* < 0.05) (Figure 7A, IBS gut/degenerative brain axis). The results shown in Figure 7B (IBS gut/degenerative brain axis) also demonstrated the ability of VitD3 NS to promote cell survival by increasing PI3K expression better than LPS 100 ng/mL + H_2_O_2_ 200 µM, VitD3 Physical Mix 100 nM, and VitD3 Sigma 100 nM (45%, 20%, and 16%, respectively, *p* < 0.05).

Afterward, further experiments were performed to check the ability of VitD3 forms to modulate the activities of both pTAU and APP. As shown in Figure 7C (IBS gut/degenerative brain axis), pTAU expression increased in the presence of LPS 100 ng/mL + H_2_O_2_ 200 µM compared with control (about 32%, *p* < 0.05). On the other hand, treatment with the different forms of VitD3 significantly reduced pTAU expression compared with LPS 100 ng/mL + H_2_O_2_ 200 µM-induced damage (*p* < 0.05). In particular, the greatest reduction was observed after treatment with VitD3 NS 100 nM (about 47% vs. LPS 100 ng/mL + H_2_O_2_ 200 µM, 25% vs. VitD3 Physical Mix 100 nM, 22% vs. VitD3 Sigma 100 nM, *p* < 0.05), indicating a beneficial effect in counteracting cognitive dysfunction. The positive effect of VitD3 NS was also supported by APP level (Figure 7D, IBS gut/degenerative brain axis); indeed, treatment with VitD3 NS 100 nM reduced the APP level about 40% vs. LPS 100 ng/mL + H_2_O_2_ 200 µM (*p* < 0.05), about 19.5% vs. VitD3 Physical Mix 100 nM (*p* < 0.05), and about 16% vs. VitD3 Sigma 100 nM (*p* < 0.05). These findings support previous findings, indicating that VitD3 NS may enhance cell survival through the IBS gut/degenerative brain axis mechanism.

Finally, the properties of VitD3 were also investigated on both BDNF and SIRT-1 as key markers of neuronal well-being. As shown in Figure 7E (IBS gut/degenerative brain axis), BDNF level was significantly increased by VitD3 NS 100 nM (*p* < 0.05) compared to LPS 100 ng/mL + H_2_O_2_ 200 µM (51%, *p* < 0.05) and the other VitD3 forms (28% vs. VitD3 Physical Mix 100 nM and 24% vs. VitD3 Sigma 100 nM, *p* < 0.05). In addition, as illustrated in Figure 7F (IBS gut/degenerative brain axis), the phosphorylation of SIRT1 induced by VitD3 NS (*p* < 0.05) was increased compared to LPS 100 ng/mL + H_2_O_2_ 200 µM (43.5%, *p* < 0.05) and the other forms of VitD3 (22% vs. VitD3 Physical Mix 100 nM and 16.5% vs. VitD3 Sigma 100 nM, *p* < 0.05), supporting the efficacy of VitD3 NS in increasing the presence of this molecule.

### 2.5. Evaluation of the Ability of VitD3 NS to Modulate Inflammation

**Figure 8:**
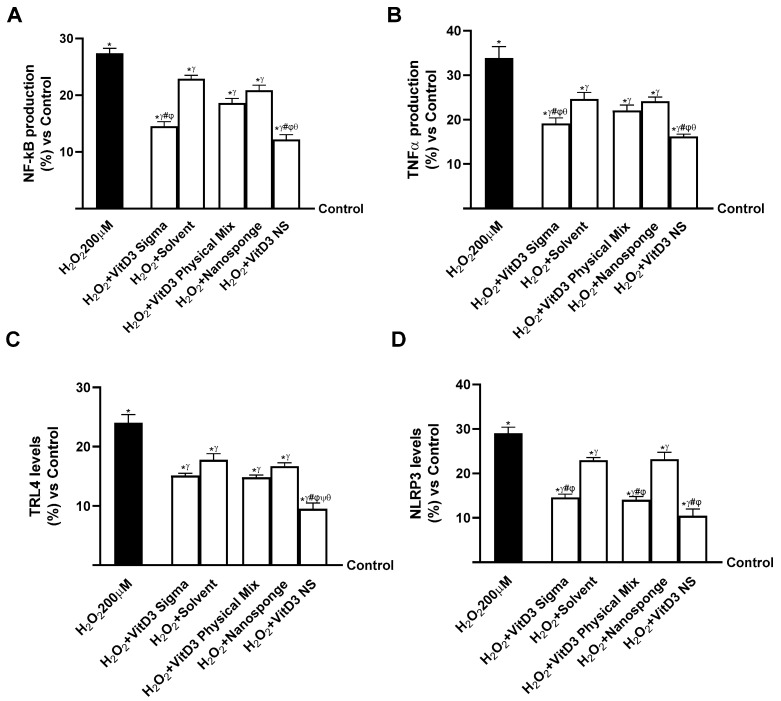
Evaluation of the ability of VitD3 NS to modulate inflammation. In (**A**), NF-kB production was measured by ELISA Kit; in (**B**), TNFα production was measured by ELISA Kit; in (**C**), TRL-4 level was measured by ELISA Kit; in (**D**), NLPR3 level was measured by ELISA Kit. Data are expressed as mean ± SD (%) of 5 independent experiments normalized to control. * *p* < 0.05 vs. control; ^γ^ *p* < 0.05 vs. H_2_O_2_ 200 µM; ^#^ *p* < 0.05 vs. solvent (ethanol); ^φ^ *p* < 0.05 vs. nanosponge; ^ψ^ *p* < 0.05 vs. VitD3 Sigma; ^θ^ *p* < 0.05 vs. VitD3 Physical Mix 100 nM.

Neuroinflammation is found to be a key phase in the neurodegenerative process as it could result in loss of synaptic function as well as lead to the onset of neurodegenerative diseases such as Alzheimer’s disease [14]. Consequently, evaluating if VitD3 was able to modulate the inflammatory condition through the healthy gut/degenerative brain axis mechanism was found to be crucial; on neuronal cells under oxidative stress conditions, the productions of NF-kB and TNFα were investigated, but also the activities of key markers of systemic inflammation such as Toll-like receptor-4 (TRL-4) and NLRP3. As shown in Figure 8A (healthy gut/degenerative brain axis), NF-kB production increased in cells treated with H_2_O_2_ 200 µM by about 27% compared to the control (0% line, *p* < 0.05). The same trend was observed in TNFα production (Figure 8B, healthy gut/degenerative brain axis) as damage increased TNFα production by about 34% compared to control (0% line, *p* < 0.05). Instead, after treatment with VitD3 NS, the production of NF-kB and TNFα was reduced by about 55% and 52% vs. H_2_O_2_ 200 µM, about 34% and 26% vs. VitD3 Physical Mix 100 nM and about 16% and 36% vs. VitD3 Sigma 100 nM, respectively. To confirm the anti-inflammatory activity of VitD3 NS, the activities of the major systemic inflammation markers TRL-4 and NLRP3 were investigated. As shown in Figure 8C (healthy gut/degenerative brain axis), TRL-4 level was decreased more after treatment with VitD3 NS compared with damage and VitD3 Physical Mix 100 nM and VitD3 Sigma 100 nM (60%, 36%, and 37%, respectively, *p* < 0.05). In addition, NLRP3 level (Figure 8D, healthy gut/degenerative brain axis) was also reduced by VitD3 NS compared with damage and other forms of VitD3 (64% vs. H_2_O_2_ 200 µM, 26% vs. VitD3 Physical Mix 100 nM, and 28% vs. VitD3 Sigma 100 nM, *p* < 0.05).

**Figure 9:**
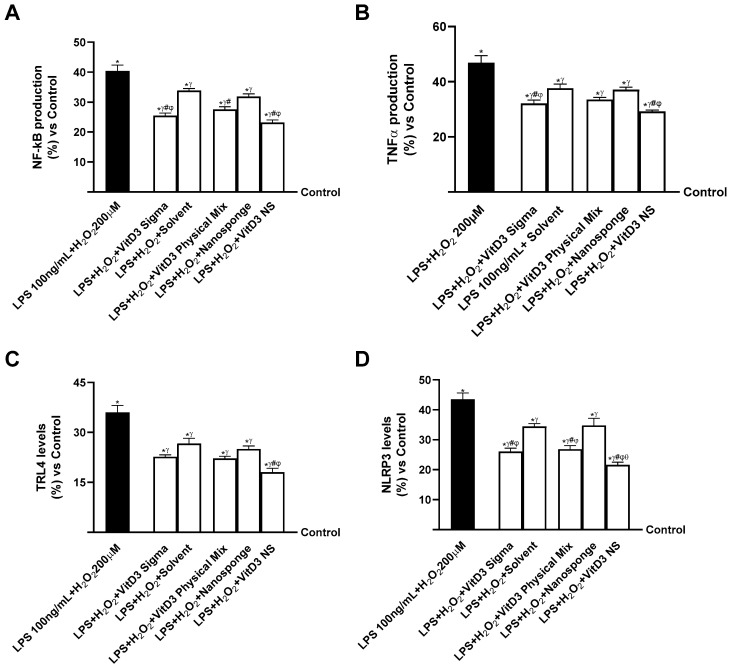
Evaluation of the ability of VitD3 NS to modulate inflammation. In (**A**), NF-kB production measured by ELISA Kit; in (**B**), TNFα production measured by ELISA Kit; in (**C**), TRL-4 level measured by ELISA Kit; in (**D**), NLPR3 level measured by ELISA Kit. Data are expressed as mean ± SD (%) of 5 independent experiments normalized to control. * *p* < 0.05 vs. control; ^γ^ *p* < 0.05 vs. LPS 100 ng/mL + H_2_O_2_ 200 µM; ^#^ *p* < 0.05 vs. solvent (ethanol); ^φ^ *p* < 0.05 vs. nanosponge; ^θ^ *p* < 0.05 vs. VitD3 Physical Mix 100 nM.

Finally, the same analysis was performed on the IBS gut/degenerative brain axis to confirm VitD3 NS anti-inflammatory properties. As reported in Figure 9A (IBS gut/degenerative brain axis), NF-kB production increased in cells treated with LPS 100 ng/mL + H_2_O_2_ 200 µM by about 40% compared to control (0% line, *p* < 0.05). The same trend was observed in TNFα production (Figure 9B, IBS gut/degenerative brain axis) as LPS 100 ng/mL + H_2_O_2_ 200 µM increased TNFα production by about 47% compared to control (0% line, *p* < 0.05). Instead, after treatment with VitD3 NS, the production of NF-kB and TNFα was reduced by about 42.5% and 37% vs. LPS 100 ng/mL + H_2_O_2_ 200 µM, about 16% and 13% vs. VitD3 Physical Mix 100 nM, and about 10% and 11% vs. VitD3 Sigma 100 nM, respectively. To confirm the anti-inflammatory activity of VitD3 NS, the activities of the major systemic inflammation markers TRL-4 and NLRP3 were investigated. As shown in Figure 9C (IBS gut/degenerative brain axis), TRL-4 levels decreased more after treatment with VitD3 NS compared with damage and VitD3 Physical Mix 100 nM and VitD3 Sigma 100 nM (50%, 18.5%, and 20%, respectively, *p* < 0.05). In addition, the NLRP3 level (Figure 9D, IBS gut/degenerative brain axis) was also reduced by VitD3 NS compared with damage and other forms of VitD3 (50% vs. LPS 100 ng/mL + H_2_O_2_ 200 µM, 19% vs. VitD3 Physical Mix 100 nM, and 17% vs. VitD3 Sigma 100 nM, *p* < 0.05).

## 3. Discussion

This study unravels the intricate interplay between VitD3 and its receptor, exploring the modulation of its biological activity through complexation with cyclodextrin nanosponges (VitD3-NS). We utilized 1,25-(OH)2-cholecalciferol, the active metabolic form of vitamin D3, leveraging cyclodextrin complexation to achieve heightened plasma levels of active vitamin D. This facilitated direct passage through the intestinal barrier and to reach the brain without requiring activation within the organism. Promising insights into its therapeutic potential along the gut–brain axis emerge from the multifaceted impact observed at both gut and brain levels [15]. VitD3-NS exhibits remarkable ameliorative effects on liposaccharide-induced damage and neurodegenerative conditions, emphasizing its enhanced efficacy. The findings open avenues for further exploration and application in diverse pathophysiological conditions.

### 3.1. Introducing the Connection between Gut–Brain Axis and Neurosteroid Dynamics of VitD3

The brain and the gut communicate via several channels within the body. In particular, the bidirectional communication system between the central nervous system and the enteric nervous system, known as the gut–brain axis, is integral to understanding the impact of nutrients, such as VitD3. Indeed, the enteric nervous system, often called the second brain, regulates digestion and gut motility, receiving signals from the autonomic nervous system. The gut–brain axis plays a pivotal role in mental comorbidities, especially in conditions like IBS, affecting 5–10% of the global population [16,17]. Furthermore, gut microbiota, influenced by factors like infectious gastroenteritis and antibiotics, contributes to IBS pathophysiology [16]. In this scenario, VitD3 has gained importance as an essential nutrient due to its chemical structure resembling a steroid hormone.

Specific VitD receptors (VitDR) are widely distributed in the brain, with the highest density in the neuroepithelium and proliferating zones [17]. The neurolocalization of VitDR, VitD3, and related enzymes in the brain categorizes it as a neurosteroid. Thus, the central nervous system is a site for both the activation and inactivation of VitD. VitD exhibits various pleiotropic activities, including the preservation of normal neural development, support for adult brain tropism, and a potential role in slowing down the aging process [18,19]. Hence, ensuring optimal VitD3 absorption efficiency is essential, particularly in gut–brain axis alterations.

### 3.2. Enhancing VitD3 Bioavailability and Efficacy Using VitD3-NS as a Novel Formulation

According to multiple research projects, VitD3 absorption efficiency ranges between 55% and 99% but is independent of dietary fat amount, while bioavailability is influenced by lipid makeup [20]. Although supplements containing liposomes, microcapsules, or VitD micelles boost vitamin D absorption efficiency, the present study introduces a unique formulation, VitD3-NS, utilizing nano-complexation with cyclodextrins (CDs) to enhance bioavailability. Indeed, CDs are one example of a unique formulation created using nanocomplexation.

Initially, we assessed the capacity of VitD3-NS to traverse the intestinal barrier in an IBS condition, aiming to confirm its ability to reach the intended target. Results from assessments of cell metabolism, oxidative stress, and lipid peroxidation revealed the favorable tolerance of VitD3-NS by intestinal cells, thereby ameliorating the IBS condition induced by LPS 100 ng/mL for 48 h.

Subsequently, a 3D model of the human intestine under IBS conditions was employed to investigate the feasibility of oral administration. The absorption study demonstrated the viability of oral delivery, with bioavailability experiments indicating effective absorption and distribution of VitD3-NS. Notably, the quantities absorbed were slightly lower than those observed in a 3D in vitro model of a healthy human intestine [5].

More specifically, analysis of the data from the healthy model revealed that VitD3-NS is present in higher concentrations upon reaching the plasma level compared to the control and two other forms of VitD3 tested, which exhibit maximum absorption at 4 h. This confirms the viewpoint that encapsulating VitD3 within the nanosponge enhances its bioavailability during the physiological timeframe of intestinal digestion, even under IBS conditions. Furthermore, VitD3-NS treatment is crucial in enhancing permeability by fortifying tight junctions (TJ). Notably, in epithelial cells, TJ formation is pivotal for maintaining the integrity of the intestinal barrier. A process mediated by essential proteins, such as claudin, occludin, and ZO-1. As anticipated, inflammatory damage induced by LPS (100 ng/mL) disrupts monolayer integrity, as evidenced by reduced TEER values and diminished claudin, occludin, and ZO-1 levels. In contrast, VitD3-NS restores epithelial integrity and facilitates ion exchanges across the intestinal barrier, indicating its capability to traverse the intestinal epithelial monolayer even under IBS conditions. Additionally, VitD3-NS reduces inflammation in the intestine, as shown by NF-kB and TNFα analysis.

In summary, these results support the idea that VitD3-NS can cross the healthy intestinal epithelium and, even under IBS conditions, reach the plasma and, consequently, the target site of VitD3 action, such as the brain. VitD3-NS’s role in enhancing intestinal integrity and ion exchange is evident, restoring epithelial function and countering LPS-induced inflammatory damage.

### 3.3. VitD3-NS and the Gut–Brain Axis: Implications for Minimal Cognitive Function and Neuroprotection

Our results underscore the need to elucidate the gut–brain axis’s role in cognitive function. We explored cognitive function using minimal cognitive function [21], which helps investigate biochemical circuits and network foundations for biological cognition in neuronal cells. To delve into this, we established an in vitro model to assess how VitD3-NS supplementation influences cognitive function mechanisms. Specifically, our experiments focused on the gut–brain axis, a bidirectional communication system connecting the central and enteric nervous systems. Using samples metabolized by intestinal cells to stimulate SHSY-5Y cells in the basolateral compartment [22], we analyzed the primary biological activities of VitD3 forms during cognitive dysfunction induced by H_2_O_2_. Importantly, the tested VitD3 types demonstrated safety, enhancing cellular antioxidant activity and reducing lipid peroxidation, with observed effects in the healthy gut/degenerative brain axis and the IBS gut/degenerative brain axis models. In both models, VitD3-NS successfully reached the target, activating VDR. However, in the IBS model, the presence of LPS 100 ng/mL damage for 48 h slightly reduced the effectiveness of VitD3-NS. To assess VitD3-NS effects on neurodegeneration, we analyzed brain markers in both gut–brain axis models. The healthy gut/degenerative brain axis model efficiently restored cell survival markers ERK/MAPKs and PI3K. Conversely, in the IBS gut/degenerative brain axis model (induced by H_2_O_2_), the restoration of these mechanisms decreased slightly compared to the damage. Additionally, pTAU and APP activities were reduced in both models, while BDNF and SIRT-1 activities were restored. Moreover, the pro-inflammatory cytokines NF-kB and TNFα, the systemic inflammation markers TRL-4 and NLRP3 were examined to assess the anti-inflammatory effect of VitD3 NS. Indeed, it has been observed that VitD3-NS can reduce the inflammatory response in the healthy gut/degenerative brain axis model and the IBS gut/degenerative brain axis model. Due to LPS 100 ng/mL-induced gut-level damage over 48 h, the effect was more evident in the healthy gut/degenerative brain axis model than in the IBS gut/degenerative brain axis model. Finally, given that the IBS condition may modify VitD3 absorption and bioavailability by lowering the amount of VitD3 that reaches the target site, the variation in the effect that VitD3 NS exerted in the two study models may be related to intestinal passage.

This study unveils the therapeutic potential of VitD3-NS along the gut–brain axis, showing efficacy in ameliorating damage and neurodegenerative conditions. VitD3-NS, with enhanced bioavailability, is promising for some pathophysiological conditions, emphasizing the importance of optimal absorption efficiency. The study suggests VitD3-NS as a potential therapeutic avenue and highlights its benefits in healthy and IBS conditions, paving the way for further exploration.

## 4. Materials and Methods

### 4.1. Agent Preparation

Anhydrous cyclodextrin and carbonyl diimidazole were kept at a 1:4 molar ratio in a solvent-free one-step reaction to create the cross-linked CD nanosponges before the preparation of the complex, as documented in the literature [23]. This was accomplished using a ball mill (Planetary Ball Mill: PM200 High-Speed Planetary Ball Mill, Retsch; Pedrengo BG, Italy). Then, 15 mg of vitamin D3 and 300 mg of NS-CDI are combined in the complex (1:4) to attain a 5% loading. According to the literature [5], thermal gravimetric analysis (TGA) was used to test its thermostability. The material was diluted for the treatments to reach a final concentration of 100 nm of Vitamin D3 [24]. VitD3 Physical Mix VitD3-BCDI 1:4 (once complexed, NS-CDI (1:4) is known as BCDI 1:4) was made by weighing 1 mg and dissolving it in 2 mL of the medium before diluting it to the final concentration to be tested in the well (100 nM). According to the literature, this concentration is best from 1 nM to 100 nM [25,26]. Cyclodextrin was chosen to combine with vitamin D3 due to its low cost, single cavity size, and widespread availability [27]. In addition, it is non-toxic, capable of forming complexes with a wide variety of molecules, and possesses an extraordinary capacity to ensnare both organic and inorganic molecules; thus, it emerges as the most suitable candidate for pursuing the objective of the present study [28]. The initial concentration of the substance was 1.3 mM when 1 mg was dissolved in 2 mL of medium. This concentration was diluted to obtain a final 100 nM [5]. The final concentrations to be evaluated (100 nM) in the well were obtained by weighing 1 mg and dissolving it in 2 mL of 100% ethanol (ET-OH, Merck Life Science, Rome, Italy). ET-OH at 0.007% was also tested to rule out vehicle interference. H_2_O_2_ 200 µM (Merck Life Science, Milan, Italy) was prepared in complete Dulbecco’s Modified Eagle Medium (DMEM; GIBCO^®^ ThermoFisher Scientific, Waltham, MA, USA) without red phenol and 0% of fetal bovine serum (FBS) to have 3.2 M which was diluted by DMEM, without red phenol to have 200 µM [29].

### 4.2. Cell Culture

The American Type Culture Collection (ATCC) human colorectal adenocarcinoma cell line CaCo-2 was cultured in Dulbecco’s Modified Eagle’s Medium Advance (Adv DMEM; GIBCO^®^ ThermoFisher Scientific, Waltham, MA, USA) supplemented with 10% fetal bovine serum (FBS), 2 mM L-glutamine (Merck Life Science, Milan, Italy), and 1% penicillin/streptomycin (Merck Life Science, Italy) and maintained at 37 °C in an incubator with 5% CO2 and 95%. Paracellular permeability and transport properties were maintained physiologically by utilizing cells with passage numbers ranging from 26 to 32 [30]. More precisely, after the cell growth reached 80% confluence, 1 × 10^4^ cells were placed in 96-well plates to investigate cell viability. This was performed using an MTT-based In Vitro Toxicology Assay Kit from Merck Life Science in Rome, Italy. The cells were synchronized by incubating them in DMEM without red phenol and supplemented with 0.5% FBS, 2 mM L-glutamine, and 1% penicillin-streptomycin at 37 °C eight hours before the stimulation. Furthermore, using ELISA kits, 1 × 10^6^ cells were cultured in 6-well plates to investigate the intracellular mechanisms. Additionally, 2 × 10^4^ cells were cultured in a 24-well plate with a 6.5-mm Transwell^®^ insert containing a polycarbonate membrane with a pore size of 0.4-μm (Corning^®^ Costar^®^, Merck Life Science, Rome, Italy) to conduct absorption analysis [31] and 2.8 × 10^5^ cells were cultured in a 6-well plate with a 24-mm Transwell^®^ insert containing a polycarbonate membrane with a pore size of 0.4-μm (Corning^®^ Costar^®^, Merck Life Science, Rome, Italy) to conduct additional absorption analysis. The cells placed on the Transwell^®^ insert were kept in a complete medium and replaced every other day on both the basolateral and apical sides for 21 days before the simulations [30]. Before stimulation, the medium on the apical side was adjusted to a pH of 6.5, equivalent to the pH in the lumen of the small intestine. Conversely, the pH 7.4 observed on the basolateral side corresponds to the acidity level of blood [32]. This in vitro model is extensively utilized [33] and endorsed by the European Medicines Agency (EMA) and Food and Drug Administration (FDA) for the prediction of the absorption, metabolism, and bioavailability of several drugs following oral ingestion in humans [34,35].

THP-1 cells, purchased from ATCC, were grown in RPMI-based CCM supplemented with 1% Penicillin/Streptomycin, 1% l-Glutamine, 1% l-Hydroxygenase, 1% d-Glucose, 1% mercaptoethanol, and 10% heat-inactivated FBS at 37 °C and 5% CO_2_. THP-1 cells were planted 3 × 10^6^ in 25 cm^2^ flasks for co-culture experiments, and they were differentiated with PMA (100 nM). After that, the cells were detached using Accutase (Merck Life Science, Rome, Italy), plated at a density of 5.3 × 10^4^ cells/well on 24-well plates and 7.5 × 10^5^ cells/well on 6-well plates that were suited for transwells, and given 1.5 h to reattach [36].

The ATCC provided the SH-SY5Y cells, which were cultivated in 1:1 Adv DMEM F12 (GIBCO^®^ ThermoFisher Scientific, Waltham, MA, USA) and Adv DMEM (GIBCO^®^ ThermoFisher Scientific, Waltham, MA, USA). Additionally, 10% fetal bovine serum (FBS, Merck Life Science, Rome, Italy) was added, along with 2 mM HEPES, 2 mM L-glutamine, and 1% penicillin/streptomycin (Merck Life Science, Rome, Italy). The cells were cultured in a 37 °C incubator with 5% CO_2_ and 95% humidity [37]. The investigations utilized cells with passage numbers ranging from 3 to 20. The cells were plated in distinct ways to conduct various experiments. For instance, to examine cell viability using an MTT-based In Vitro Toxicology Assay Kit (Merck Life Science, Rome, Italy), Cytochrome C to quantify ROS production and lipid peroxidation analysis, 8 × 10^4^ cells were plated on 24-well plates. Before the stimulation, the cells were synchronized by incubating them for eight hours in Adv DMEM (GIBCO^®^ ThermoFisher Scientific, Waltham, MA, USA) without red phenol. To further support the cells, 0.5% FBS (Merck Life Science, Rome, Italy), 2 mM L-glutamine, and 1% penicillin–streptomycin were added to the medium at 37 °C. In addition, the cells were plated at 4 × 10^5^ cells in 6-well plates to study the inflammatory markers such as NF-kB, TNFα, TRL-4, and NLRP3 and intracellular pathways involved, including ERK, PI3K, pTAU, APP, BDNF, and Sirt-1 using an ELISA kit.

### 4.3. Experimental Protocol

In the study, three previously tested forms of Vitamin D3 were used [5] to evaluate their effect at the central nervous system level following intestinal passage. More specifically, two gut–brain axes models were constructed, one with a healthy bowel and the other with an inflamed version, to assess whether the effect on the final target could be modified based on the intestinal condition. The experiments were divided into two steps; the first one aimed to verify the ability of three different forms of Vitamin D3 to cross the healthy and inflamed intestinal barrier in vitro model excluding negative effects, and the second one was to check the direct effects on neuronal cells analyzing several parameters and mechanism of actions. To validate the previous findings regarding ROS production and cell viability, intestinal CaCo-2 cells were subjected to treatment with a Physical Mix containing Vitamin D3-BCDI 1:4 loaded nanosponge (referred to as VitD3 Physical Mix), BCDI (1:4 nanosponge, referred to as nanosponge), and reference VitD3. This treatment was known as VitD3 NS. Following that, an intestinal in vitro barrier model was utilized to evaluate these compounds to ascertain intestinal integrity via tight junction analysis (ELISA kit), permeability assay (Papp measurement), and TEER measurement (TEER), as well as to determine the total quantity of VitD3 that crossed the intestinal barrier. As documented in the literature [5,32], cells were subjected to time-dependent treatments ranging from 1 to 6 h for each experiment. The same experiments were performed on an inflamed intestinal barrier in vitro model generated after the 48 h pretreatment with LPS 100 ng/mL of the cell co-culture of CaCo-2 and THP-1 [36]. In addition, after each stimulation, the basolateral medium was collected to treat neuronal cells for 24 h after pre-treatment with H_2_O_2_ 200 µM for 30 min. At the end of the stimulation, the cell viability, ROS production, NO production, and Lipid peroxidation were analyzed. In addition, the inflammatory markers such as NF-kB, TNFα, TRL-4, and NLRP3 and intracellular pathways involving ERK, PI3K, pTAU, APP, BDNF, and Sirt-1 were measured using an ELISA kit.

### 4.4. Cell Viability

Following the manufacturer’s instructions, the In Vitro Toxicology Assay Kit (Merck Life Science, Rome, Italy) [5] was used to analyze cell viability using a traditional MTT-based approach. After all treatment, the cells were allowed to incubate for two hours at 37 °C, 5% CO_2_, and 95% humidity with 1% MTT dye. The purple formazan crystals were dissolved in an equivalent amount of MTT Solubilization Solution. A spectrophotometer (infinite 200 Pro MPlex, Tecan, Männedorf, Switzerland; Tecan) was used to measure the absorbance at 570 nm with a correction at 690 nm. The results were expressed to the control (0% line), representing untreated cells.

### 4.5. In Vitro Intestinal Barrier

An intestinal barrier model using CaCo-2 and PMA-differentiated THP-1 cells was performed to analyze the passage through the intestinal barrier of three different forms of Vitamin D3, having, as a final destination, the neuronal cells where they could exert their beneficial effects. The TEER values were measured using EVOM3 and STX2 chopstick electrodes from World Precision Instruments, located in Sarasota, FL, USA. This assessment was conducted every 2 days over 21 days until a TEER value of ≥400 Ωcm^2^ was achieved, indicating the formation of a cell monolayer, cell differentiation, and exposure of intestinal villi [36,38]. On the 21st day, the medium in the apical and basolateral environments was altered to provide distinct pH conditions: approximately 6.5 pH at the apical level (resembling the acidic pH of the small intestinal lumen) and approximately 7.4 pH at the basolateral level (resembling the neutral pH of human blood) [32]. For the co-culture to be defined as stable, it is good to know that changes in the growth environment may cause a temporary reduction in TEER of ~10% compared to the control CaCo-2 monoculture in the first 24 h of co-culture. After that, the TEER value should recover above 95% after 24 h of co-culture. In addition, co-culture should not induce macrophage activation or stimulate intestinal epithelial cells (IECs) by preventing the release of pro-inflammatory cytokines in the basolateral compartment from significantly exceeding the levels of cytokines released by THP-1 monocultures without stimulation. The TEER values were tested again before the experiment started to confirm the results’ stabilization after the cells were maintained for 15 min at 37 °C and 5% CO2. The cells were exposed to three distinct formulations of VitD3 for a duration ranging from 2 to 6 h before subsequent analysis. This analysis included the measurement of permeability using Papp analysis [5,32]. Concisely, the Papp (cm/s) was computed using the subsequent formula:

Papp = dQ/dt ⇥ 1/m0 ⇥ 1/A ⇥ V Donor

dQ: amount of substance transported (nmol or μg);
dt: incubation time (sec);
m0: amount of substrate applied to donor compartment (nmol or μg);
A: surface area of Transwell membrane (cm^2^);
VDonor: volume of the donor compartment (cm^3^).

Negative controls without cells were tested to exclude Transwell membrane influence.

### 4.6. TJs Analysis

The CaCo-2 lysates were used to analyze occludin level by Human Occludin (OCLN) ELISA Kit (MyBiosource, San Diego, CA, USA), claudin-1 level by ELISA Kit (Cusabio Technology LCC, Huston, Houston, TX, USA), and Zona Occludens 1 (ZO-1) level by the human tight junction protein 1 (TJP1) ELISA kit (MyBiosource, San Diego, CA, USA) following the manufacturer’s instructions [31]. The absorbance was measured by a Tecan spectrophotometer at 450 nm. The data were obtained by comparing it to the standard curve (from 0 to 1500 pg/mL for occludin and from 0 to 1000 pg/mL for both claudin-1 and ZO-1). The results were presented as a percentage (%) compared to the control (0 line), which comprised five separate experiments conducted in triplicate.

### 4.7. Vitamin D Quantification

The metabolically active form of vitamin D was detected using the competitive ELISA assay kit (FineTest), as documented in the literature [29]. Specifically, following each treatment, a volume of 50 μL was collected from each sample and promptly used. During each sample, 50 μL of biotin-detection and 100 μL of SABC working solution were introduced and incubated at 37 °C for 30 min. Finally, the liquid portion above the sediment was removed, and then 90 μL of TMB substrate and 50 μL of stop solution were introduced. Subsequently, the 96-well plate underwent analysis using a Tecan spectrometer set at a wavelength of 450 nm. Furthermore, it was important to construct a standard curve including a range of 1.563–100 ng/mL, which also incorporated the background (zero well) to quantify it.

### 4.8. VDR Assay Kit

The presence of the Human Vitamin D Receptor (VDR) in cell lysates of the co-culture was measured using the VDR ELISA kit (MyBiosource, San Diego, CA, USA), following the instructions provided by the manufacturer. In summary, the cells were disrupted using trypsin and subsequently gathered using centrifugation. After that, the cells were rinsed three times with cold PBS 1× and then suspended in PBS 1×. Next, the cells underwent ultrasonication four times, followed by centrifugation at 1500× *g* for 10 min at 4 °C to eliminate cellular debris. Then, 100 μL of each sample was added to a well and incubated at 37 °C for 90 min. Afterward, the materials were withdrawn, and 100 μL of Detection Solution A was added to each well and incubated for 45 min at 37 °C. After washing the wells with Wash Solution, 100 μL of Detection Solution B was applied to each well. Following a 45-min incubation period, the wells were rinsed once more. Subsequently, 90 μL of Substrate Solution was introduced into each well and incubated for an additional 20 min at 37 °C while being kept in darkness. Then, 50 μL of Stop Solution was introduced, and the plates were analyzed using a Tecan spectrometer at a wavelength of 450 nm. The concentration was quantified in nanograms per milliliter (ng/mL), and the obtained results were compared to the standard curve, which spanned a range from 0.625 ng/mL to 40 ng/mL.

### 4.9. ROS Production

Superoxide anion release was quantified using a standard methodology based on cytochrome C reduction [5,32]. A Tecan spectrophotometer was used to detect the absorbance in culture supernatants at 550 nm after adding 100 μL of cytochrome C (Merck, Milan, Italy) to each well. Comparatively, empty wells were filled with 100 μL of superoxide dismutase (Merck, Milan, Italy) and 100 μL of cytochrome C, and the plate was incubated for 30 min. The O_2_ rate was quantified as the average standard deviation (%) of nanomoles per decreased cytochrome C per microgram of protein relative to the control (0 line).

### 4.10. NO Production

To quantify nitrogen oxide (NO) production, a kit was used on SHSY-5Y cell supernatants, following the manufacturer’s instructions [39]. The absorbance of the samples was read with a Tecan spectrometer at a wavelength between 520 nm and 550 nm. The results are expressed as mean ± SD (%) compared to the untreated control sample, normalized to the standard curve generated by the standard nitrate.

### 4.11. Lipid Peroxidation Assay

The TBARS Assay Kit (Cayman Chemical, Tallinn, Estonia) was used to measure the amount of lipid peroxidation in cells [40]. In summary, 100 μL of sample or standard was mixed with 100 μL of sodium dodecyl sulphate (SDS) solution. Subsequently, a volume of 4 mL of Dye Reagent was introduced into each vial, followed by a boiling process lasting for 1 h. Next, 150 μL of samples or standard were added to each well of a 96-well plate after the samples had cooled for 10 min. The absorbance of each sample was measured using a Tecan spectrometer at 530–540 nm. Vials were centrifuged at 1600× *g* for 10 min at 4 °C. Analysis compared results to untreated cells (0% line) and a standard curve (0–50 µM).

### 4.12. ERK/MAPKS ELISA Kit

The InstantOneTM ELISA (Thermo Fisher, Milan, Italy) measured ERK/MAPK expression in SHSY-5Y lysates [41]. After treatment, cells were lysed with 100 μL Cell Lysis Buffer Mix, agitated for 10 min at room temperature, and analyzed in 50 μL/well InstantOne ELISA microplate strips. After adding 50 μL of the antibody cocktail to each well and letting it sit for one hour at room temperature on a microplate shaker, wash the wells three times using 200 μL of Wash Buffer for each run. Finally, 100 μL of Detection Reagent and 100 μL of Stop Solution were added to each well to halt the reaction after 20 min. The strips were measured using Tecan at 450 nm. The results were measured by quantifying the average absorbance (%) to the control.

### 4.13. Human Phosphotylinosital 3 Kinase (PI3K) ELISA Kit

PI3K expression in the cell supernatant was measured using an ELISA kit (Human Phosphotylinosital 3 kinase (PI3K) ELISA Kit, MyBiosource, San Diego, CA, USA) according to standard technique [42]. Sample absorbance was measured at 450 nm using a Tecan spectrophotometer. Results were compared to the standard curve (range from 2 to 600 ng/mL) and expressed as mean SD (%) normalized to the control value (line 0).

### 4.14. BDNF Quantification ELISA Kit

Human BDNF Elisa Kit (Thermo Scientific^TM^, Waltham, MA, USA) quantified BDNF in SHSY-5Y cell supernatants following the manufacturer’s instructions. In summary, a detection antibody labeled with biotin was introduced into each well, and the plate was left to incubate at room temperature for 1 h. After 45 min of HRP-conjugated streptavidin incubation, TMB substrate solution was added for 30 min, and a Stop Solution terminated the reaction. The BDNF concentration was measured using a Tecan spectrometer at 450 nm and compared to the standard curve (0.066 to 16 ng/mL) [32]. Results are shown as mean ± SD (%) compared to the untreated control sample. The cells were also given 10 ng/mL BDNF as a positive control [43].

### 4.15. SIRT-1 Assay Kit

Quantification of SIRT1 protein was measured with SIRT1 ELISA Kit (Thermo Scientific^TM^, Waltham, MA, USA) on SHSY-5Y cell lysate. Briefly, 100 μL of each sample was added and incubated at 37 °C for 90 min; then, the material was removed, and 100 μL of Detection Solution A was added and incubated for 45 min at 37 °C. After this, the wells were washed, and 100 μL of Detection Solution B was added to each well and then incubated for 45 min at 37 °C. Then, 90 μL of substrate solution was added to each well, and the plate was incubated for 20 min at 37 °C in the dark. Next, 50 μL of Stop Solution was used to stop the reaction. The concentration was reported as ng/mL compared to a reference curve (range from 1.23 to 300 ng/mL) [44]. The absorbance was measured using a Tecan spectrometer at 450 nm. The findings are presented as mean SD (%) to the control group (line 0).

### 4.16. Amyloid Precursor Protein (APP) Assay Kit

The Amyloid Beta A4 protein ELISA kit (Merck Life Science, Milan, Italy) assessed APP in SHSY-5Y cell supernatants following manufacturer instructions. Cellular supernatants were collected and analyzed with an ELISA kit after treatment completion. The Biotinylated Detection Antibody, specific to the target protein, was introduced into each well. Subsequently, the plate was incubated at room temperature for 1 h. Following a 45-min incubation period with HRP-conjugated streptavidin, the TMB substrate solution was introduced for 30 min. Finally, the reaction was halted by adding the Stop Solution. The concentration of APP was measured using a Tecan spectrometer by determining the absorbance at 450 nm. The concentration was computed by comparing the results to the APP standard curve, which ranged from 0.1 to 100 ng/mL [29]. The data are presented as mean ± SD (%) compared to the untreated control sample.

### 4.17. Human Tau (Pospho) Protein Assay Kit

The Tau (Phospho) [pS199] Human ELISA Kit (Thermo Scientific^TM^, Waltham, MA, USA) was used to measure Tau protein in SHSY-5Y cell lysates. Add 100 μL of each sample to the plate, incubating at 37 °C for 90 min. Remove the material and wash the wells three times. Detection Solution A (100 μL) was applied to each well and incubated at 37 °C for 45 min. After incubation, wash wells, add 100 μL Detection Solution B, and incubate at 37 °C for 45 min. After adding 90 μL of Substrate Solution, the plate was incubated at 37 °C for 20 min in the dark. The reaction was stopped with 50 μL of Stop Solution, and the absorbance was measured at 450 nm using a Tecan spectrometer. Data are expressed in ng/mL based on the standard curve (15.6–1000 pg/mL), and findings are shown as mean SD (%) relative to the control (line 0) [45].

### 4.18. NF-kB Analysis

Following the manufacturer’s instructions, the NF-kB (p65) Transcriptional Factor Assay kit was used to measure DNA binding activity [32]. Results were compared to the standard curve (NF-kB (p65) Transcriptional factor positive control) to determine the concentration (range from 0 to 10 µL/well with scaled dilutions) and reported as means ± SD (%) relative to control (0 lines).

### 4.19. TNFα Assay Kit

Following the manufacturer’s instruction, TNFα production on the SHSY-5Y cell line under oxidative stress conditions was analyzed by the Human Tumor Necrosis Factor α ELISA Kit (Merck Life Science, Rome, Italy). Next, 100 µL of material was placed in each well of a 96-well ELISA plate, incubated at room temperature for 2 h, and overnight at 4 °C. Five washings with washing buffer followed incubation, and adding 100 μL of biotinylated anti-TNFα to each well. After a 2-h room temperature incubation, each well was aspirated and cleaned five times before adding 100 μL Streptavidin–HRP for a 1-h incubation. Incubate 100μL of chromogen solution in each well for 30 min at room temperature in darkness after washing the plate with Streptavidin–HRP solution. The absorbance of each well was measured at 450 nm using a Tecan plate reader after applying Stop Solution [41]. The data are presented as mean SD (%) relative to the untreated control sample (0% line).

### 4.20. Human TRL-4 ELISA Kit

TRL-4 protein was investigated by the Human TRL-4 ELISA Kit (Thermo Scientific^TM^, Waltham, MA, USA) on SHSY-5Y cell lysates. Briefly, 100 μL of each sample was added to the plate and incubated at 37 °C for 90 min; then, the material was removed, and the wells were washed three times. Then, add 100 μL of Detection Solution A to each well and incubate for 45 min at 37 °C. After incubation, wash wells, add 100 μL Detection Solution B, and incubate at 37 °C for 45 min. After adding 90 μL of Substrate Solution, the plate was incubated at 37 °C for 20 min in the dark. A Tecan spectrometer assessed absorbance at 450 nm after stopping the reaction with 50 μL of Stop Solution. The concentration is expressed in ng/mL by comparing the data with the standard curve (range from 0.4 to 100 ng/mL), and the results are expressed as mean ± SD (%) compared with the control (line 0).

### 4.21. Human NLRP3 ELISA Kit

Following manufacturer directions, cell lysates were tested using the Human NLRP3 ELISA Kit (Abcam, Cambridge, UK). After overnight incubation at 4 °C, 100 μL of diluted samples were washed four times with 1× Wash Buffer. Then, 100 μL of detection antibody was added to each well and incubated for 1 h at room temperature with gentle shaking. After washing the wells four times, incubate the plate with 100 μL of streptavidin–HRP for 45 min. Following incubation, wells were rinsed, and 100 μL of TMB substrate was added. After 30 min of incubation at room temperature with gentle shaking, the reaction was stopped using 50 μL of Stop Solution. The Tecan spectrometer measured the absorbance at a wavelength of 450 nm. The absorbance values were then converted to ng/mL using a standard curve that ranged from 4.38 to 280 ng/mL. The results were given as the mean ± SD expressed as a percentage relative to the control. The experiments were conducted five times independently, each completed in triplicate.

### 4.22. Statistical Analysis

Results come from at least four independent experiments per protocol, with averages ± SD based on three technical replicates. Statistical analysis included one-way ANOVA, Bonferroni post hoc test, Mann–Whitney U tests, and Welch’s test for pairwise differences. Statistical significance was defined as *p* < 0.05.

## 5. Conclusions

In conclusion, this study demonstrates for the first time the ability of cyclodextrin-nanosponge complexes to improve the biological function and bioavailability of VitD3 in the intestine. Specifically, its composition confirms the safety through an in vitro study on intestinal cells under LPS-induced IBS. This confirms the potential of VitD3 complex as a novel dietary supplement. In addition, the efficacy of the complex was further confirmed through a two-axis gut–brain study, both physiologically and under IBS conditions; specifically, the VitD3 NS complex was able to exert its neuroprotective functions counteracting degenerative process induced in the brain by H_2_O_2_. While the in vitro data are very clear and promising, in vivo or even human studies would be needed to confirm these observations before assuming the absolute efficacy of this complex. Therefore, the results of the present study on the efficacy in improving VitD3 absorption may support the hypothesis that oral administration in humans can be considered a viable therapeutic strategy to achieve beneficial therapeutic effects under conditions of low VitD3.

## Data Availability

Raw data are preferably deposited at the Laboratory of Physiology UPO and at the Dept. Di Chimica IFM University of Turin, ensuring appropriate measures so that raw data are retained in full forever under a secure system. The data presented in this study are available on reasonable request from the corresponding author.

## Abbreviations

Adv DMEM	Advance Dulbecco’s Modified Eagle’s Medium
Adv DMEM/F12	Advanced Dulbecco’s Modified Eagle Medium F12
ANOVA	Analysis of Variance
APP	β-amyloid
ATCC	American Type Culture Collection
CDs	Cyclodextrins
FBS	fetal bovine serum
GBA	Gut–Brain Axis
IBS	inflammatory bowel syndrome
LPS	lipopolysaccharide
MTT	3-(4,5-Dimethylthiazol-2-yl)-2,5-diphenyltetrazolium bromide
NS	Nanosponge
OCLN	Occludin
Papp	Apparent permeability coefficient
PBS	Phosphate Buffered Saline
ROS	Radical oxygen species
SD	standard deviation
SDS	Sodium dodecyl sulfate
SOD	Superoxide Dismutase
TBARS	Thiobarbituric acid reactive substances
TEER	Transepithelial electrical resistance values
TGA	Thermal gravimetric analysis
TJ	Tight Junction
TJP1	Human Tight Junction Protein 1
TRL-4	Toll-like receptor-4 TRL-4
VDR3	Vitamin D Receptor
VitD3	Vitamin D3
VitD3 NS	Vitamin D3-BCDI 1:4 nanosponge
ZO-1	Zona occludens 1
